# The effects of 28‐day early‐life exposure to triphenyl phosphate (TPhP) on odor preference and sexual behavior in female rats

**DOI:** 10.1002/jat.4021

**Published:** 2020-06-22

**Authors:** Airi Nakayama, Tatsuya Hattori, Anna Isobe, Shohei Kobayashi, Go Suzuki, Hidetaka Takigami, Maiko Kawaguchi

**Affiliations:** ^1^ Laboratory of Animal Behavior and Environmental Science, Graduate School of Agriculture Meiji University Kawasaki Kanagawa Japan; ^2^ Organization for the Strategic Coordination of Research and Intellectual Property Meiji University Kawasaki Kanagawa Japan; ^3^ Center for Material Cycles and Waste Management Research National Institute for Environmental Studies Ibaraki Japan

**Keywords:** early‐life exposure, female rats, flame retardant, odor preference, Oral administration, sexual behavior, Triphenyl phosphate

## Abstract

Many chemical substances are detectable in house dust, and they are consequently taken into our bodies via the mouth and nose. Triphenyl phosphate (TPhP), a flame retardant that has an estrogen‐like effect in vitro, is present in house dust at high concentrations. Estrogen exposure during development has significant influences on reproductive behavior in rodents, and its effects persist until maturity. In the present study, we investigated the effect of early life exposure to TPhP on the reproductive behavior of female rats. Oral treatment with TPhP (25 or 250 mg/kg), ethinyl estradiol (EE; 15 μg/kg) as a positive control, or sesame oil as a negative control, were given to female rats (from birth to 28 days of age). The 8‐week‐old rats were bilaterally ovariectomized. At 12–15 weeks of age, the rats were subjected to odor preference and sexual behavior tests. In the odor preference test, the oil group showed significantly higher preference for male odor than female odor, but the low‐dose TPhP treatment group lost the preference for male odor, indicating a possible outcome of early life TPhP exposure on sexual recognition. In the sexual behavior test, both the EE and TPhP treatment groups displayed significantly less proceptive behavior. These results suggest that early life exposure to TPhP disturbs the normal sexual behavior of female rats.

## Abstract 2

Triphenyl phosphate (TPhP) is a flame retardant detected in house dust at high concentrations. TPhP exhibits estrogenic effect in vitro; however, its effect remains unclear in vivo. We examined the effects of early life exposure to low (25 mg/kg) and high (250 mg/kg) doses of TPhP in female rats on reproductive behavior. In the case of sexual behavior, TPhP significantly reduced proceptive behavior. This study showed that early life exposure to TPhP disturbs the normal sexual behavior of female rats.

## 1 INTRODUCTION

Although early mammalian brains are sexually undifferentiated, there is an important period in the developmental process where they are highly susceptible to sex hormones. The brain differentiates into male form when exposed to androgens of testicular origin in this critical period, whereas sex hormones are considered unnecessary for differentiation in females. The perinatal period is the most critical period in rodents, which is from the late fetal stage to the immediate postnatal stage. Moreover, the effects of hormonal exposure on sexual differentiation in the brain are permanent, affecting adult partner preference (Henley, Nunez, & Clemens, 2009) and sex‐specific sexual behaviors (Monje, Varayoud, Muñoz‐de‐Toro, Luque, & Ramos, 2009).

Estrogen is more important than androgens for sexual differentiation in small rodent brains (Maekawa, Tsukahara, Kawashima, Nohara, & Ohki‐Hamazaki, 2014). When testosterone in the blood reaches the brain, it is converted to estradiol by aromatase, which acts by binding to estrogen receptors. Perinatal estrogen and environmental estrogenic substances have been suggested to have a serious effect on rodent brain development and sexually dimorphic behavior. For example, when bisphenol A, an estrogenic substance, was administered to female rats from postnatal days (PNDs) 1–7, there was no effect on lordosis or ear wiggling after maturation, but hopping and darting decreased significantly (Monje et al., 2009). Vaginal opening and periodicity were also delayed, and ovarian dysfunction occurred, following a single β‐estradiol 3‐benzoate (EB; 10 μg) administration immediately after birth, causing a decrease in lordosis and a decrease in temptation behavior (Berretti et al., 2014). Furthermore, female rats given an implant (EB‐containing silicone tube) in their backs on PND 0 showed a preference for females over males in partner preference tests (Henley et al., 2009).

House dust is dust floating in the living space of animals, including humans. Many chemical substances are detected in house dust, and they are taken into our body orally, dermally, and through inhalation. Flame retardants are one class of chemical substances detected in house dust, which are widely used in the manufacture of plastics, woods, fibers, and gums. They are also applied to furniture to prevent the spread of flames in the event of a fire. Flame retardants are classified as either brominated or phosphorous flame retardants. Polybrominated diphenyl ethers (PBDEs), a type of brominated flame retardant, are widely used, although they are regulated or prohibited from use due to their health effects (Darnerud, Eriksen, Jóhannesson, Larsen, & Viluksela, 2001). Accordingly, phosphorous flame retardants have been increasingly used instead of PBDEs in recent years (Meeker, Johnson, Camann, & Hauser, 2009).

Triphenyl phosphate (TPhP) is one of the flame retardants detected in house dust at high concentrations. In fact, TPhP has been detected in 98% of house dust samples and reported at concentrations of 173–1800 ng/g (John & Heather, 2010). TPhP has estrogen effects in vitro (Suzuki et al., 2013). In in vivo studies, young male Sprague–Dawley rats were fed diets containing TPhP at levels of 0–1.0% for 4 months, and their behavior was tested, including motility and exploratory behavior assessment (open field test), balance and general motor coordination testing (rotarod test), and muscular strength measurement (forelimb grip test) (Sobotka, Brodie, Arnold, West, & O'donnell, 1985). There was no effect on nerve/muscle function or general condition in all groups; however, a significant inhibition of body weight gain was observed in the group administered with ≥0.5% TPhP (Sobotka et al., 1985). Moreover, when young male and female Sprague–Dawley rats were fed diets containing TPhP at levels of 0–1.0% for 120 days, there was a clear dose‐dependent inhibition of body weight gain during the first 2 months, and body weight gain at 0–4 weeks in the 1% TPhP group was significantly lower in male rats (Hinton, Jessop, Arnold, Albert, & Hines, 1987). There was a significant increase in serum β‐globulin in males at ≥0.35% TPhP and in serum α‐globulin in females, although there was no influence on liver and thymus weights; spleen, thymus, and mesenteric lymph node tissues; and immune response (Hinton et al., 1987). Lastly, when young male and female Sprague–Dawley rats were fed diets containing TPhP at levels of 0–1.0% for 91 days, administered through the mating period until the 20th day of pregnancy, the body weight of the females in the 1% TPhP group was significantly lower on day 0 of pregnancy, and body weight gain (except the pregnant uterus) also tended to be low in the group treated with ≥0.5% TPhP (Welsh, Collins, Whitby, Black, & Arnold, 1987). In contrast, there was no effect on lutein levels implantation rate, number of surviving fetuses, number of dead fetuses, incidence of malformations, and mutations in all groups (Welsh et al., 1987). In addition to these studies using rats, TPhP is even detectable in the human body (Kim et al., 2014), facilitating better understanding of the effects of TPhP on the animal body.

In our laboratory, we showed that 28‐day early‐life exposure to TPhP in male rats resulted in inhibition of development of the accessory gland and penis, and partially suppressed sexual behavior after maturation (unpublished data). However, there is no study investigating the effects of TPhP exposure on early postnatal female rats. Furthermore, no study has investigated TPhP on female reproductive behavior from a behavioral point of view. In the present study, we focused on the estrogenic action of TPhP and demonstrated the effects of early life exposure to TPhP, or ethinyl estradiol (EE) as a positive control, for estrogen‐like action on odor preference (presumably related with partner preference; Xiao, Kondo, & Sakuma, 2004) and sexual behavior in female rats.

## 2 MATERIALS AND METHODS

### 2.1 Animals

In all, 19 pregnant female Wistar–Imamichi rats (9‐, 10‐, and 12‐days pregnant) were purchased from the Institute for Animal Reproduction (Kasumigaura, Ibaraki, Japan) and individually housed in polycarbonate cages (200‐mm wide × 410‐mm long × 250‐mm high) until parturition. On the day of parturition (PND 0), the number of offspring was adjusted to eight regardless of sex. All offspring were weaned on PND21 and kept with two to four rats per cage with same‐sex littermates, but only female offspring were used in the following experiments. A total of 152 female rats were randomly assigned to four treatment groups [oil, *n* = 32; low‐dose TPhP (LTP), *n* = 40; high‐dose TPhP (HTP), *n* = 40; and EE, *n* = 40]. Male rats of the same strain as the female rats were purchased from the Institute for Animal Reproduction and housed in polycarbonate cages, with two to four rats per cage.

All the rats were maintained in an air‐conditioned animal room (temperature, 24 °C ± 2 °C; relative humidity, 55% ± 15%; and 12‐h light/dark schedule with lights on from 10:00 to 22:00 hours) with food (MF; Oriental Yeast Co., Tokyo, Japan) and water available ad libitum. The animal procedures were reviewed and approved by the Animal Care and Use Committee of Meiji University (#IACUC14–0002).

### 2.2 Treatments

Oral treatments of LTP (25 mg/kg; Cas‐No. 115–86‐6, >99% Tokyo Chemical Industry, Tokyo, Japan), HTP (250 mg/kg), EE (15 μg/kg; Cas‐No. 57–65‐6, >98%, Tokyo Chemical Industry) as the positive control (Ryan, Hotchkiss, Crofton, & Gray, 2009), or the same volume of sesame oil (5 mL/kg; Cas‐No. 8008‐74‐0, Sigma‐Aldrich, St. Louis, MO, USA) as the negative control, were given to female rats (aged 0–28 days). The chemical substrate doses were chosen because they had been shown to influence behavioral expression in a pilot study. Oral administration via gavage was conducted once daily until PND28 for a total exposure period of 28 days. This duration was based on Test Guideline 407 from the Organization for Economic Cooperation and Development for repeated‐dose toxicity test.

### 2.3 Ovariectomy and hormone priming

At 8 weeks of age, all females in all experimental groups were bilaterally ovariectomized under isoflurane (Cas‐No. 26675–46‐7, Mylan, Inc., Osaka, Japan) anesthesia (2–2.5%). These ovariectomized rats were allowed to recover for at least 3 weeks after surgery and were then treated with 5 μg EB (Cas‐No. 200–043‐7, >98% Sigma‐Aldrich) at 48 h and with 500 μg progesterone (Cas‐No. 57–83‐0, >98%, Wako Chemical, Co., Ltd., Tokyo, Japan) at 3–4 h prior to behavioral testing for induction of estrus.

### 2.4 Odor preference test

**FIGURE 1:**
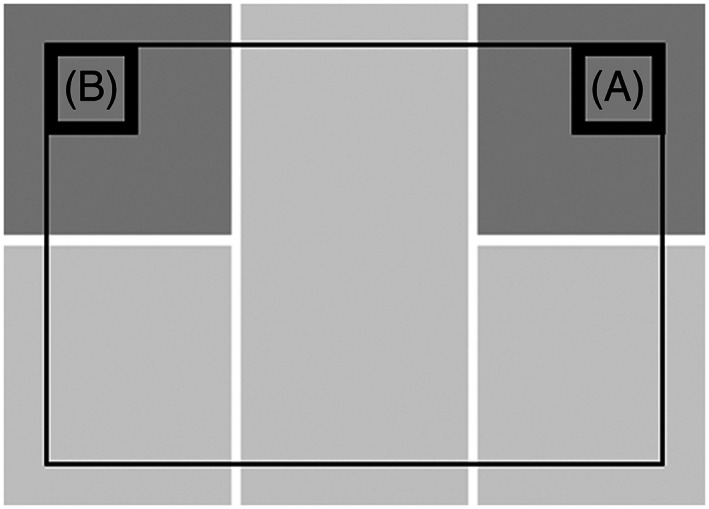
Area division within the odor preference test chamber. Before the introduction of the test rats, soiled beddings from males and females were placed in area A or B

From 12 to 15 weeks of age, the rats were tested under red lamp illumination (10–18 lux). All tests were performed between 15:00 and 17:00 hours. Soiled bedding from males and females were placed in the test chamber (700‐mm wide × 400‐mm long × 400‐mm high; Figure 1) before the test rats were introduced. The duration of time spent by the rats in the soiled bedding from male and female areas was measured.

### 2.5 Sexual behavior test

After the odor preference test, the rats were tested for sexual behavior before and after copulation experience. Male rats of the same strain and age as the female rats were allowed to practice copulation three times, and those individuals that showed sexual behavior were used subsequently. Different male rats were used for the first and second tests, and all data were analyzed. All tests were performed between 15:00 and 19:00 hours. The female rat was moved from its home cage into the test cage (700‐mm wide × 290‐mm long × 300‐mm high) 3 min before the introduction of the male rat. All behavioral tests were video recorded for subsequent analysis of behavioral categories by two observers who were not aware of the group parameters. Proceptive behaviors were measured by the frequency and latency of ear wiggling (rapid lateral shaking of the head, which produced distinct vibratory movements of ears) and hopping (jumping in front of the male rats). The receptivity behavior of each female rat was determined by using the lordosis quotient (LQ; number of lordosis/numbers of mounts × 100). Additionally, rejection responses (active resistance to a mounting attempt that resulted in the apparent abortion of the mount, such as escaping, rising on her hind legs, and kicking) were recorded. The following parameters were recorded: (1) ear wiggling latency, (2) hopping latency, (3) lordosis latency, (4) rejection latency, (5) ear wiggling number, (6) hopping number, (7) LQ, and (8) rejection number.

### 2.6 Data analysis

Data were analyzed using the IBM Statistical Package for the Social Sciences (SPSS®) software, version 22 (IBM Corp., Armonk, NY, USA). In the odor preference test, behavior was analyzed using Student’s *t*‐test. Kaplan–Meier survival analysis, followed by a log‐rank comparison test, was used to analyze latency to the first expression of mounting, ear wiggling, hopping, lordosis, and rejection. In the sexual behavior test, each behavior parameter was analyzed using one‐way ANOVA according to Tukey–Kramer. Data are expressed as the mean ± SEM, and *P* < 0.05 was considered statistically significant.

## 3 RESULTS

### 3.1 The effects of early life exposure to TPhP on odor preference in adult female rats

**FIGURE 2:**
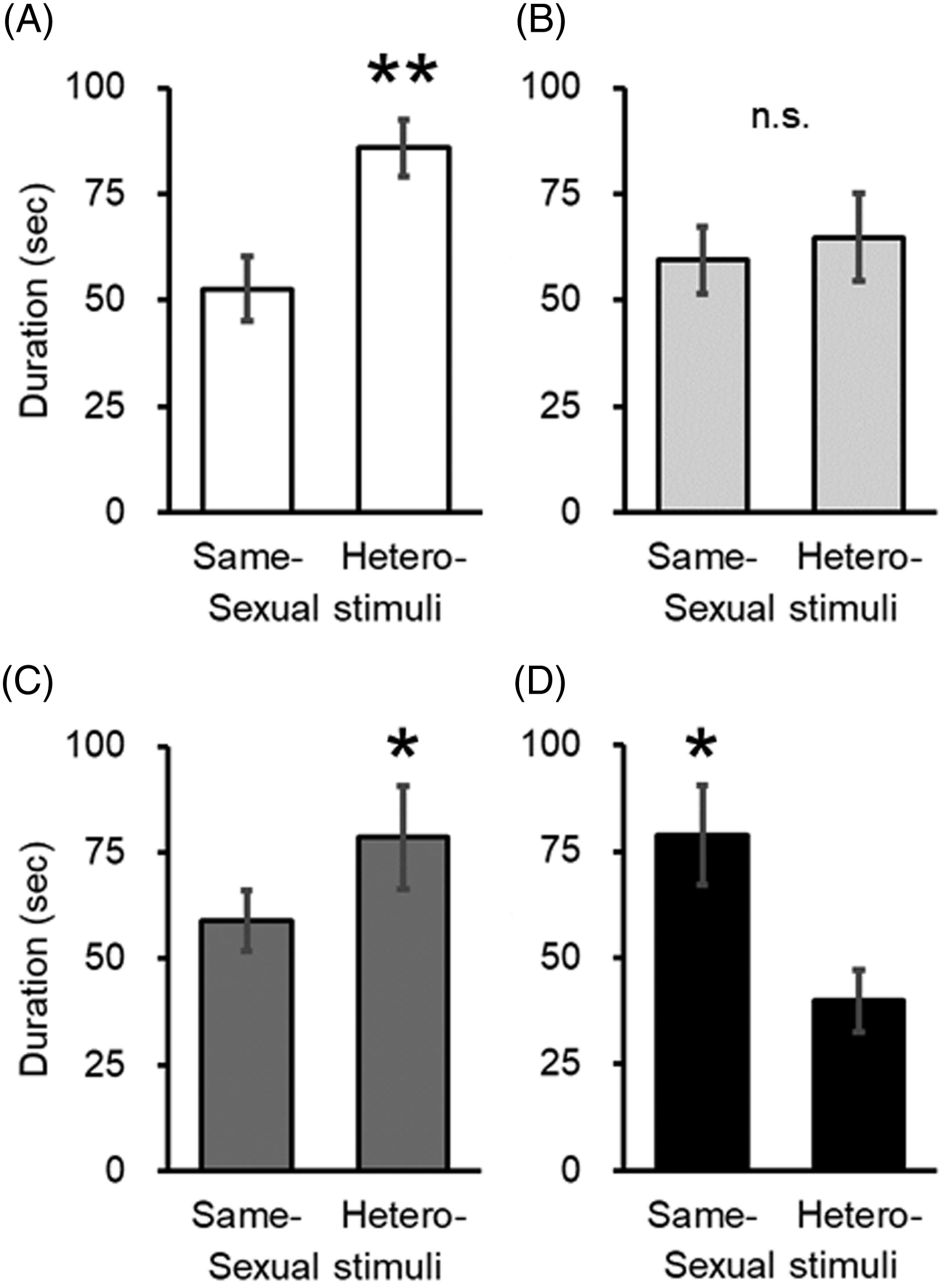
TPhP exposure impacted female odor preference in a dose‐dependent manner. The time spent for approaching the female and male bedding stimuli for the (A) oil, (B) low‐dose TPhP (LTP), (C) high‐dose TPhP (HTP), and (D) ethinyl estradiol (EE) groups. Differences between each column: **, *P* < 0.01; *, *P* < 0.05; n.s, not significant. Data are represented as mean ± SEM

We performed odor preference tests using male and female bedding as sexual stimuli to assess if TPhP impacted female sexual recognition. The results for the oil and HTP groups showed that the time spent in the male bedding area was significantly longer than that in the female bedding area [oil, t(7) = 4.08, *P* < 0.01; HTP, t(8) = 2.37, *P* < 0.05; Figure 2]. In the LTP group, the female rats interestingly spent equal time in the female and male bedding areas [t(7) = 0.13, *P* = 0.89]. However, in the EE group, the time spent in the male bedding area was significantly shorter [t(6) = 2.48, *P* < 0.05].

### 3.2 The effects of early life exposure to TPhP on sexual behavior in adult female rats

**FIGURE 3:**
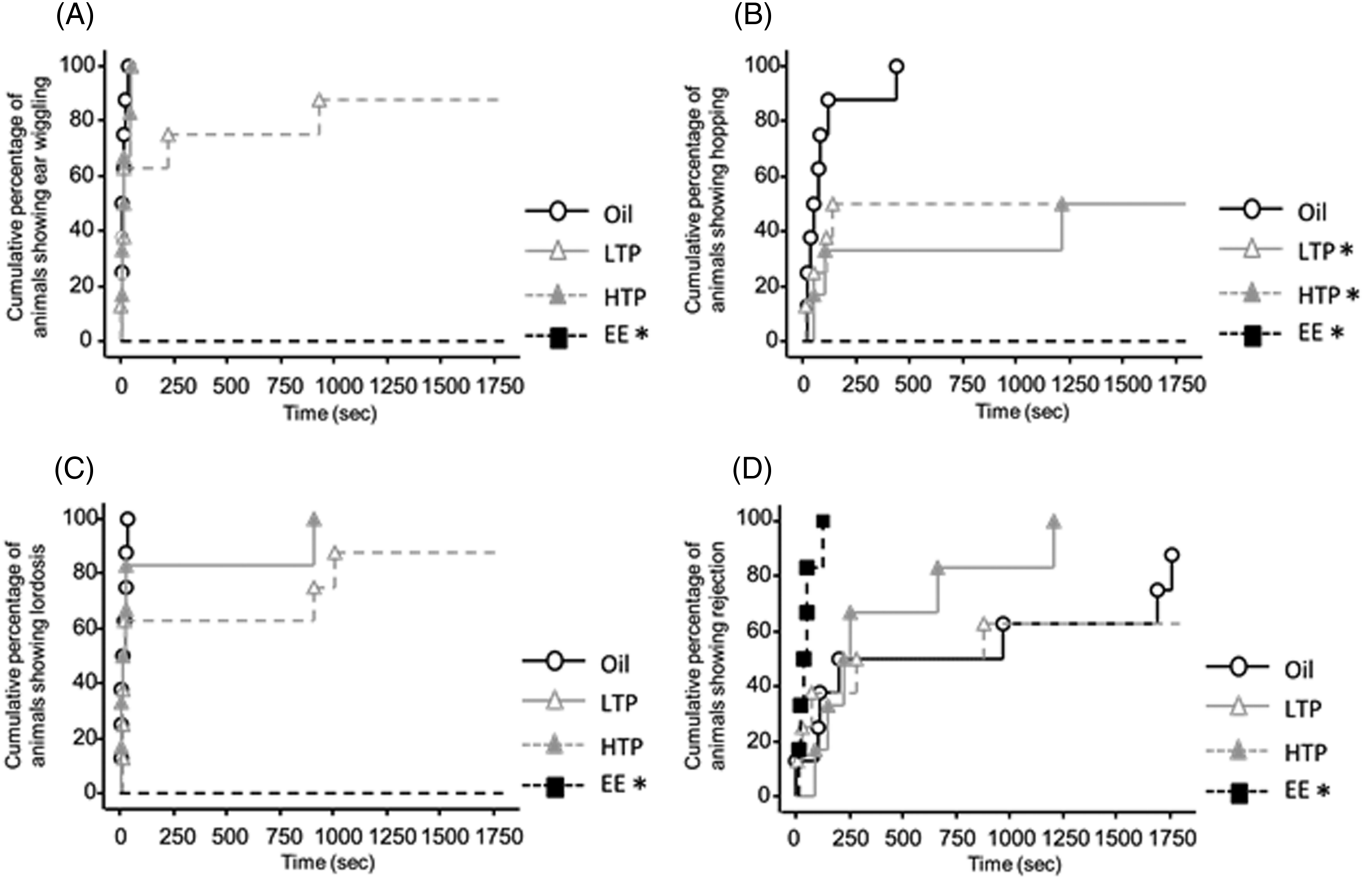
Latency to (A) first ear wiggling, (B) first hopping, (C) first lordosis, and (D) first rejection in the sexually naïve tests. *Significantly different from the oil group (*P* < 0.05). LTP, low‐dose TPhP; HTP, high‐dose TPhP; EE; ethinyl estradiol. Data are represented as mean ± SEM (oil: *n* = 8, LTP: *n* = 8, HTP: *n* = 6, EE: *n* = 6)

Latency times to the onset of sexual behavior in sexually naïve males are shown in Figure 3. Latency time to the onset of ear wiggling was longer in the EE group than that in the oil group (log‐rank test, *P* < 0.05; Figure 3A). Latency time for hopping was longer in the LTP, HTP, and EE groups than that in the oil group (log‐rank test, *P* < 0.05; Figure 3B). Latency time to the onset of lordosis was longer in the EE group than that in the oil group (log‐rank test, *P* < 0.05; Figure 3C). Latency time to the onset of rejection was shorter in the EE group than that in the oil group (log‐rank test, *P* < 0.05; Figure 3D).

**FIGURE 4:**
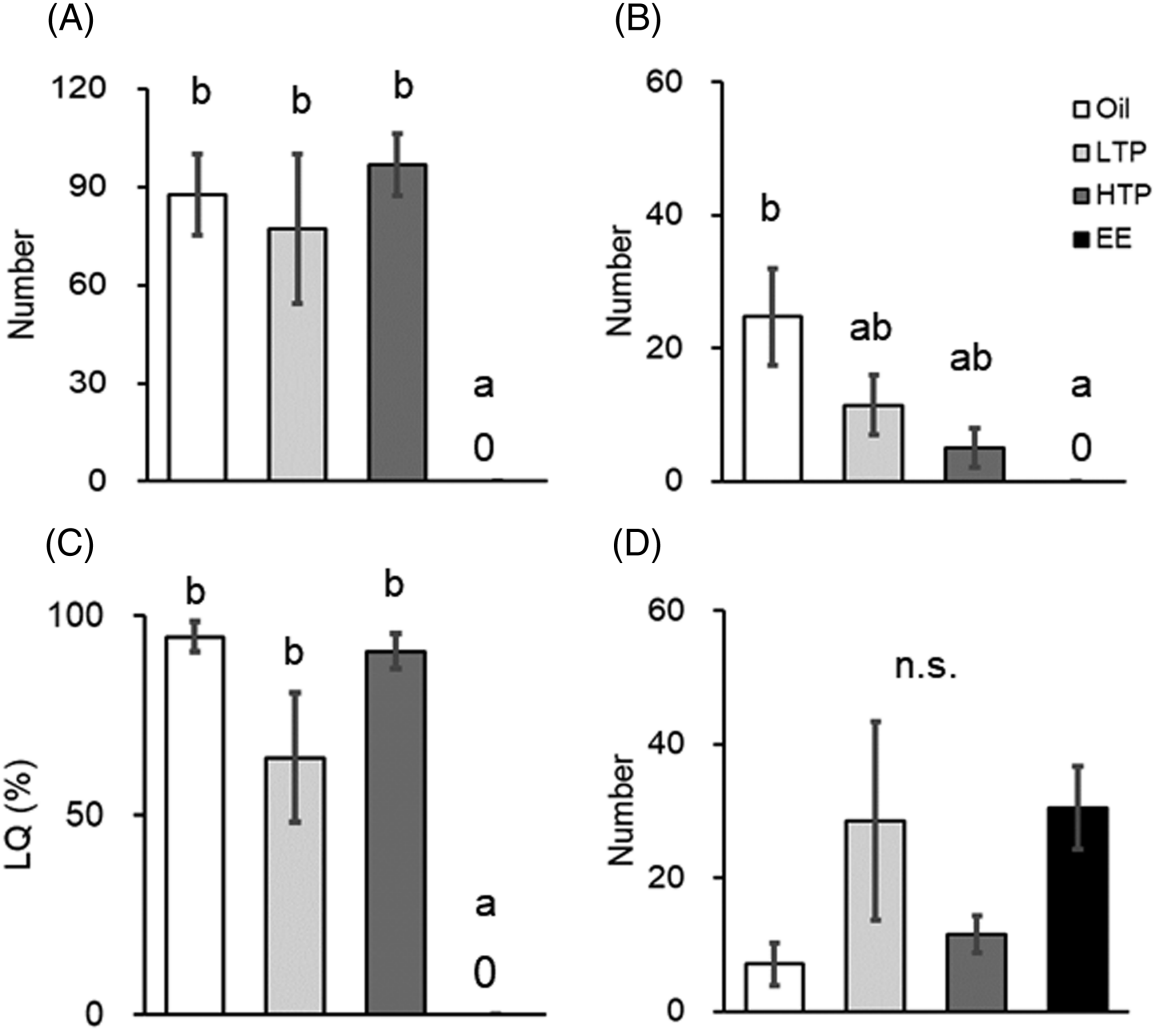
Number of (A) ear wiggling, (B) number of hopping, (C) lordosis quotient (LQ), and (D) rejections in the sexually naïve tests. Different lower‐case letters (a vs. b) indicate significant differences (*P* < 0.05). LTP, low‐dose TPhP; HTP, high‐dose TPhP; EE, ethinyl estradiol. Data are represented as mean ± SEM (oil: *n* = 8, LTP: *n* = 8, HTP: *n* = 6, EE: *n* = 6)

The numbers of sexual behaviors in sexually naïve males are shown in Figure 4. One‐way ANOVA revealed a significant group effect for ear wiggling [F(3, 24) = 7.253, *P* < 0.05], hopping [F(3, 24) = 4.445, *P* < 0.05], and LQ [F(3, 24) = 18.339, *P* < 0.05], whereas for rejection, there was no significant difference [F(3, 24) = 1.663, *P* = 0.201]. The subsequent Tukey HSD tests demonstrated that ear wiggling, hopping, and LQ were significantly reduced in the EE group (Figure 4A–C, *P* < 0.05) when compared with those in the oil group.

**FIGURE 5:**
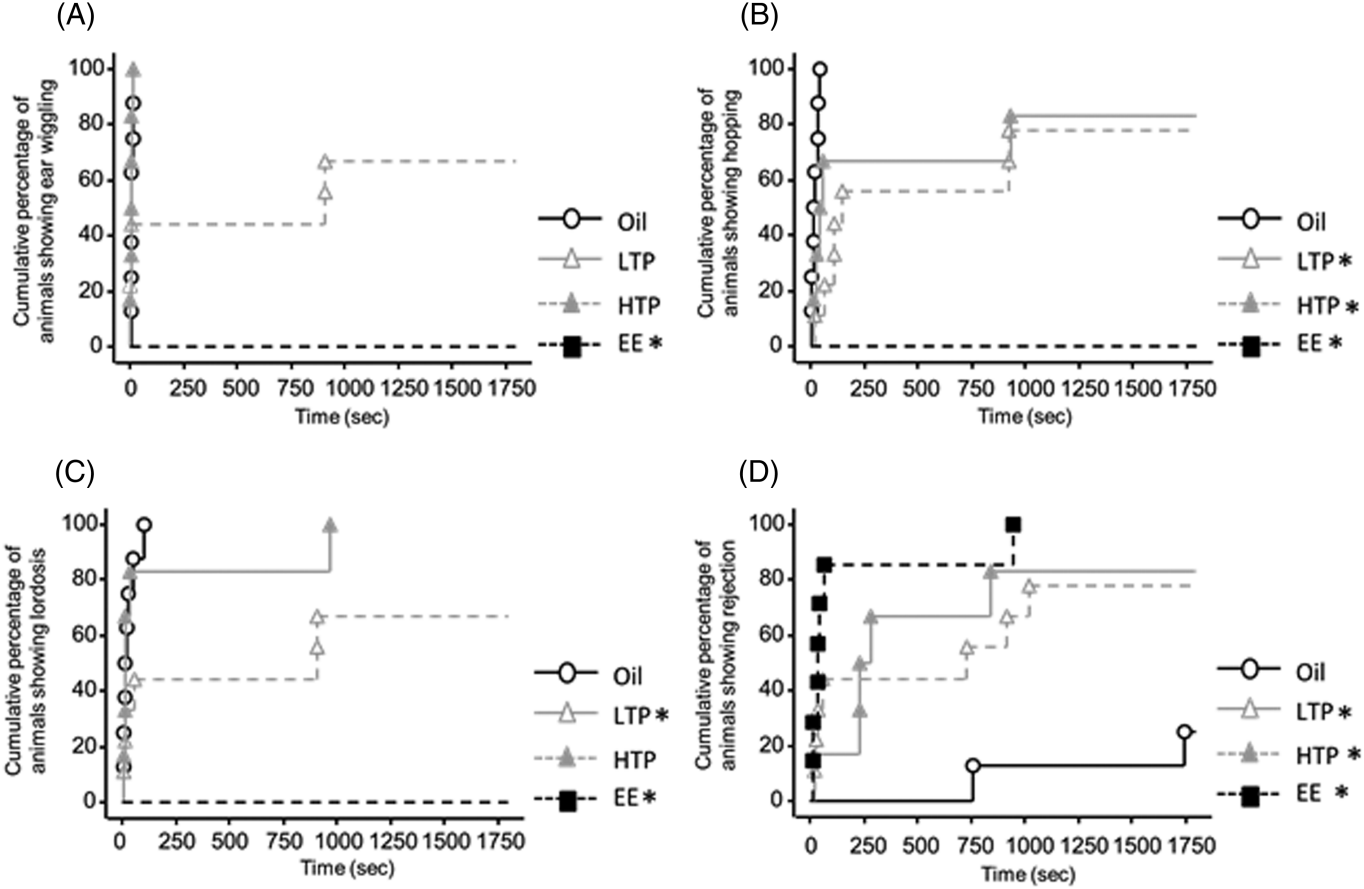
Latency to (A) first ear wiggling, (B) first hopping, (C) first lordosis, and (D) first rejection (D) in the sexually experienced tests. * Significantly different from the oil group (*P* < 0.05). LTP, low‐dose TPhP; HTP, high‐dose TPhP; EE, ethinyl estradiol. Data are represented as mean ± SEM (oil: *n* = 8, LTP: *n* = 9, HTP: *n* = 6, EE: *n* = 7)

Latency times to the onset of sexual behavior in sexually experienced males are shown in Figure 5. Latency time to the onset of ear wiggling was longer in the EE group than that in the oil group (log‐rank test, *P* < 0.05) (Figure 5A). Latency time to the onset of hopping was longer in the LTP, HTP, and EE groups than that in the oil group (log‐rank test, *P* < 0.05; Figure 5B). Latency time to the onset of lordosis was longer in the LTP and EE groups than that in the oil group (log‐rank test, *P* < 0.05) (Figure 5C). In addition, latency time to the onset of rejection was shorter in the LTP, HTP, and EE groups than that in the oil group (log‐rank test, *P* < 0.05; Figure 5D).

**FIGURE 6:**
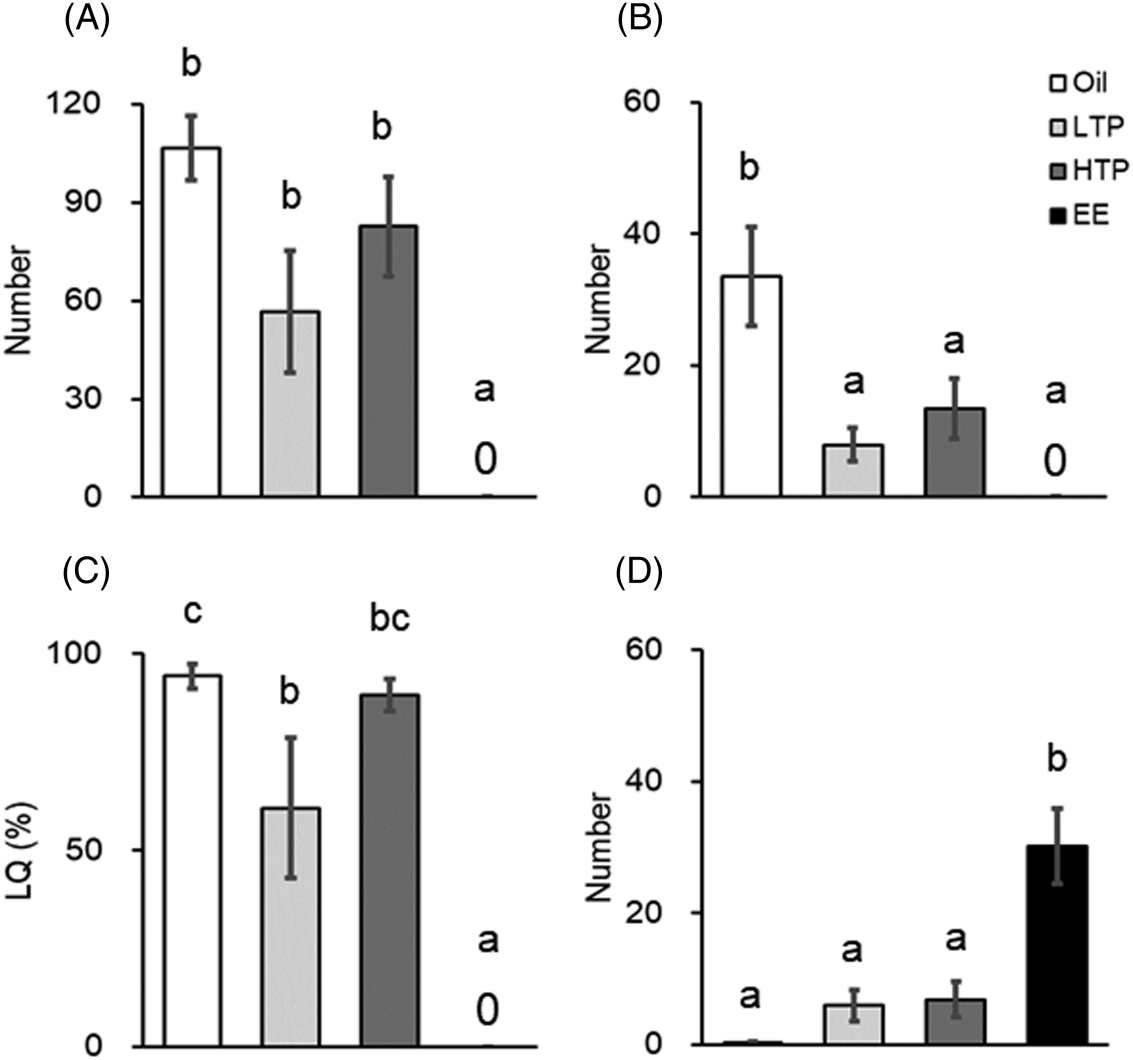
Number of (A) ear wiggling, (B) number of hopping, (C) lordosis quotient (LQ), and (D) rejections in the sexually experienced tests. Different lower‐case letters (a vs. b vs. c) indicate significant differences (*P* < 0.05). LTP, low‐dose TPhP; HTP, high‐dose TPhP; EE, ethinyl estradiol. Data are represented as mean ± SEM (oil: *n* = 8, LTP: *n* = 9, HTP: *n* = 6, EE: *n* = 7)

The numbers of sexual behaviors in sexually experienced males are shown in Figure 6. One‐way ANOVA revealed a significant group effect for ear wiggling [F(3, 26) = 10.596, *P* < 0.05], hopping [F(3, 26) = 9.631, *P* < 0.05], LQ [F(3, 26) = 15.742, *P* < 0.05], and rejection [F(3, 26) = 16.308, *P* < 0.05]. Subsequent Tukey HSD tests demonstrated that (1) ear wiggling was significantly reduced in the EE group (Figure 6A, *P* < 0.05); (2) hopping was significantly reduced in the LTP, HTP, and EE groups (Figure 6B, *P* < 0.05); (3) LQ was significantly reduced in the LTP and EE groups (Figure 6C, *P* < 0.05); and (4) rejection was significantly increased in the EE group (Figure 6D, *P* < 0.05), when compared with those in the oil group.

## 4 DISCUSSION

In the present study, we evaluated whether TPhP acts as an estrogen function disruptor and impacts female sexual recognition, as TPhP has estrogenic activity in vitro (Suzuki et al., 2013). To investigate this, we performed odor preference tests using male and female bedding as sexual stimuli. The results showed that although there was no same effect between the TPhP and EE groups, at least, both the LTP and EE groups lost odor preference toward the hetero‐sex (ie, male), indicating a possible outcome of early life TPhP exposure on sexual recognition.

There was no difference in male mounting behavior among the groups in the sexual behavior tests (data not shown). Therefore, it appears that male behavior was not changed by the approach of the females. Moreover, the EE group showed a similar effect for LQ as described previously (Ryan et al., 2009), revealing that the positive control was effective in the present study. The other measures, such as ear wiggling, hopping, and time spent by female rats in the male bedding area were also effective in the EE group. Therefore, from these results, we thought there was no problem with the administration of the experiments.

In the present study, the females spent equal time between the female and male bedding areas in the odor preference test, LQ declined, and there was an extension of lordosis latency in the sexual behavior test in the LTP group but not in the HTP group. This may be explained by an “inverted‐U” effect in the toxicity test. In toxicity tests, the harmful effects of a chemical substance often tend to become stronger as the dose increases. However, it has been clarified that substances that are suspected as endocrine‐disrupting chemicals may not necessarily have stronger adverse effects with dose increments (Kundakovic et al., 2013; Peluso, Munnia, & Cappi, 2014; Timms et al., 2005; Weiss, 2011). Moreover, our previous study showed that in female rats exposed to EE immediately after birth, avoidance learning was altered in the low‐concentration EE exposure group but not in the high‐concentration EE exposure group (Shiga et al., 2016). In the present study, we considered that the decrease in LQ with LTP may be due to TPhP exhibiting an inverted U‐shaped dose–response curve.

Estrogen receptor α (ERα) is greatly involved in the control of sexual behavior in females, because spontaneous and receptive sexual behavior decreased markedly in ERα‐deficient animals, as was previously reported (Ogawa et al., 1998). Specifically, female sexual behavior is influenced by ERα and progesterone receptors in the hypothalamic regions, such as the medial preoptic nucleus (MPN) and the ventromedial nucleus (VMH) (Monje et al., 2009). Monje et al. (2009) showed that bisphenol A, an estrogen‐like substance that was given to female rats in the perinatal period, affected sexual behavior by suppressing ERα expression in the MPN and VMH. Therefore, suppression of the spontaneous behaviors of the LTP and HTP groups in the present study may have been caused by a reduction in ERα levels in the MPN and VMH because of the administration of an estrogenic substance.

Additionally, regarding female proceptive behavior, the hopping latency was different between the TPhP and oil groups, whereas ear wiggling latency was not. This result that only hopping was affected by estrogen‐like substances was consistent with that of other studies (Komine et al., 2017; Monje et al., 2009). Therefore, hopping may be susceptible to developmental estrogenic substance exposure, which suggests that the active sexual behaviors in female rats are thought to differ in sensitivity to estrogen‐like substances.

Saito, Onuki, and Seto (2001) reported that the maximum air concentration of TPhP in houses and office buildings in Tokyo was 15.1 and 13.5 ng/m^3^, respectively. In addition, the maximum value of TPhP contained in the outer casings of computer monitors and television sets were 20.7 and 6.7 g/m^2^/h, respectively, and the median values were 0.69 and 0.33 g/m^2^/h (Saito, Onuki, & Seto, 2006), respectively. Moreover, TPhP was found in grain [26.1 ng/g of wet weight (ww)], cheese (10.9 ng/g of ww), mussels (6.3 ng/g of ww), and vegetables (2.0 ng/g of ww) in Belgian foodstuffs (Poma et al., 2018). The median level of TPhP contained in human breast milk was 1.4 ng/g lipid weight (Kim et al., 2014). The results from the present study show that at the concentrations used, TPhP may influence the normal development of female rodents. It should be noted that, the concentrations of TPhP used in the present study (25 and 250 mg/kg) are notably much higher levels than expected to be contained in the environment we live in. Therefore, the effect‐triggering TPhP concentrations that are expected to be orally ingested daily are still unknown. Further investigations are needed to see whether the TPhP concentrations in the environment affect the sexual and soliciting behavior of rats.

## CONFLICT OF INTEREST

The authors declare no conflict of interest.
